# Optimal Control Predicts Human Performance on Objects with Internal Degrees of Freedom

**DOI:** 10.1371/journal.pcbi.1000419

**Published:** 2009-06-26

**Authors:** Arne J. Nagengast, Daniel A. Braun, Daniel M. Wolpert

**Affiliations:** 1 Computational and Biological Learning Lab, Department of Engineering, University of Cambridge, Cambridge, United Kingdom; 2 Department of Experimental Psychology, University of Cambridge, Cambridge, United Kingdom; 3 Bernstein Center for Computational Neuroscience, Albert-Ludwigs Universität Freiburg, Freiburg, Germany; Northwestern University, United States of America

## Abstract

On a daily basis, humans interact with a vast range of objects and tools. A class of tasks, which can pose a serious challenge to our motor skills, are those that involve manipulating objects with internal degrees of freedom, such as when folding laundry or using a lasso. Here, we use the framework of optimal feedback control to make predictions of how humans should interact with such objects. We confirm the predictions experimentally in a two-dimensional object manipulation task, in which subjects learned to control six different objects with complex dynamics. We show that the non-intuitive behavior observed when controlling objects with internal degrees of freedom can be accounted for by a simple cost function representing a trade-off between effort and accuracy. In addition to using a simple linear, point-mass optimal control model, we also used an optimal control model, which considers the non-linear dynamics of the human arm. We find that the more realistic optimal control model captures aspects of the data that cannot be accounted for by the linear model or other previous theories of motor control. The results suggest that our everyday interactions with objects can be understood by optimality principles and advocate the use of more realistic optimal control models for the study of human motor neuroscience.

## Author Summary

Humans are highly skilled at tool use. Simple tools have no internal degrees of freedom. For example, knowing the position and orientation of a hammer allows us to, in theory, predict the forces it will generate on our hand when we wield it and the consequences our actions will have on the hammer. In contrast, more complex tools can have internal degrees of freedom, such as a glass of water in which the motion of the fluid (the internal degree of freedom) is not fully determined by the current position and orientation of the glass. Such objects can be difficult to control. Here we use a robotic interface to simulate complex objects with internal degrees of freedom and find that subjects are able to learn to control the objects and that the pattern of movement found across subjects is similar. We develop an optimal feedback control model and explain complex object interactions as a simple trade-off between effort and task accuracy.

## Introduction

Humans regularly interact with objects with internal degrees of freedom from carrying a glass of water to using a cloth to polish a table. While objects with no internal degrees of freedom can be regarded as a fixed extension of our limbs [1],[2] non-rigid objects pose a more complex control problem. The state of the object can often only be influenced indirectly and with a significant time delay, and requires the acquisition of an internal model of the object's dynamics [3],[4]. We are relatively experienced with simple objects of this class such as carrying a cup of coffee. In contrast, more complex objects with internal degrees of freedom can be highly counterintuitive to manipulate and it may take a long time to learn how to control them, for example when using a lasso. Recently, stochastic optimal feedback control has emerged as a normative framework for human motor coordination [5]–[7]. Given the dynamics and noise characteristics of our limbs, an optimal behavior can be computed that optimizes certain criteria such as a trade-off between effort and positional accuracy. Optimal control theory has been used to explain average movement trajectories as well as trial-by-trial variability in a wide range of motor behaviors, such as obstacle avoidance [8] and bimanual coordination [9]. General principles of human movements have since emerged from this framework such as the minimum-intervention principle [7]. However, the interaction with objects with internal degrees of freedom has not been investigated. We conducted a set of experiments, in which subjects had to manipulate objects with complex and unusual dynamics that were non-intuitive to control. We extended the optimal control framework to such object manipulation with internal degrees of freedom, in which both the position of the hand and the object need to be controlled. Unlike hand-held rigid objects, for which there is a one-to-one correspondence between the state of the hand and the object, for objects with internal degrees of freedom this is no longer true. In addition to the standard optimal control model, in which the hand is modeled simply as a point mass, we also used a more realistic optimal control model, which included the dynamics of a two-link arm [10]–[12]. We show that the trajectories and velocity profiles we observed experimentally could be explained by a simple cost function and that the more realistic optimal control model captures aspects of the data that the point-mass model cannot explain. Furthermore, we tested our model on data from a previous study [13] and show that our optimal control model can also account for the experimental data of a relatively simple mass-spring object.

## Results

**Figure 1:**
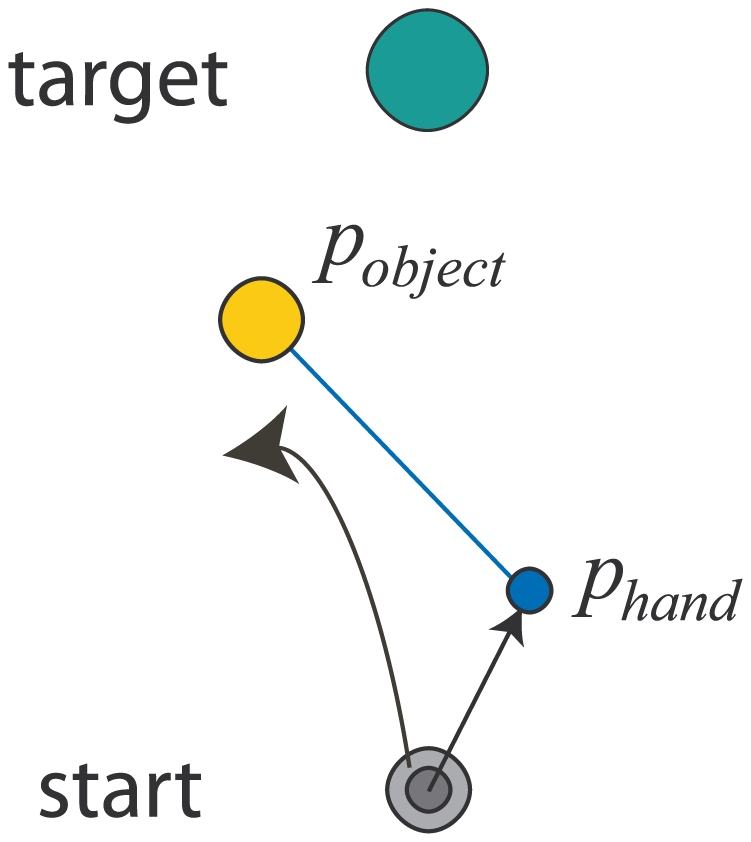
Schematic of the task. Subjects were required to move both their hand (represented by the blue circle) and object (represented by the yellow circle) to a target (green circle) within a time window which was reduced to 0.8−1.2 s over the course of the experiment. The slack length of the spring (blue line) was zero and subjects started each trial with both their hand and the object at the same position within the start region (small and large grey circles represent the initial hand and object positions). During the trial the hand and object position differed substantially due to the complex dynamics of the mass-spring-damper system. To finish a trial successfully both the hand and object had to be on the target with their speed below a threshold of 0.1 ms^−1^ (low damping conditions) and 0.02 ms^−1^ (high damping conditions).

Subjects manipulated 6 different objects with internal degrees of freedom that were simulated in a virtual-reality setup. Subjects held the handle of a robotic interface that was free to move in the horizontal plane. Their hand was attached to a virtual mass (the object) by a spring (Figure 1). The hand, mass and spring were displayed and the task required subjects to move both their hand and the mass from a start position to a target region within a certain time limit. The robotic interface was used to simulate complex dynamics of the hand-object interaction. The position of the object 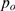 was updated based on the hand's position 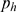 according to 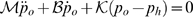 where 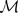, 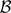 and 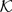 are the mass, damping and spring matrices (2×2) respectively. For a standard physical system these would be diagonal matrices but to examine the learning of complex objects we included off-diagonal terms in addition to the standard diagonal terms (see Methods for details).

The objects were non-intuitive to control and subjects had no prior experience of their dynamics. For example, the inclusion of off-diagonal terms in 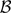 (condition B) meant that the object experienced an additional force that was orthogonal to its movement direction and proportional to its speed. This is a similar field to the standard velocity-dependent curl field [14]–[16] but with the forces applied to the object rather than to the hand directly. Inclusion of off-diagonal terms in 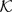 (condition K) meant that the spring also applied forces orthogonally to its stretch direction and the forces scaled in proportion to the stretch. Inclusion of off-diagonal terms in 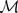 (condition M) meant that forces applied to the object caused an acceleration of the object orthogonal to the force. These three object characteristics were all paired with either low damping (conditions B-low, K-low & M-low with small diagonal terms in 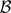) or with high damping (conditions B-high, K-high & M-high large diagonal terms in 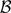).

Subjects first received a training session with a progressively stricter time criterion to facilitate learning and to familiarize them with the dynamics of the objects. To assess stable performance subjects had to continue the test session until they reached a performance criterion of 25% trials achieved within a time constraint. All six subjects achieved this for all objects although some took more trials than others (Table 1).

**Table 1:** Test sessions to criterion.

	B-low	B-high	K-low	K-high	M-low	M-high	sum
**Subject 1**	1	1	1	1	1	1	**6**
**Subject 2**	1	4	1	1	1	4	**12**
**Subject 3**	1	1	1	1	4	1	**9**
**Subject 4**	2	1	6	2	3	1	**15**
**Subject 5**	1	3	4	3	1	1	**13**
**Subject 6**	1	1	1	1	1	2	**7**
**Sum**	**7**	**14**	**11**	**11**	**9**	**10**	**62**

Number of 200 trial test sessions that subjects required to reach the criterion of 25% correct trials.

**Figure 2:**
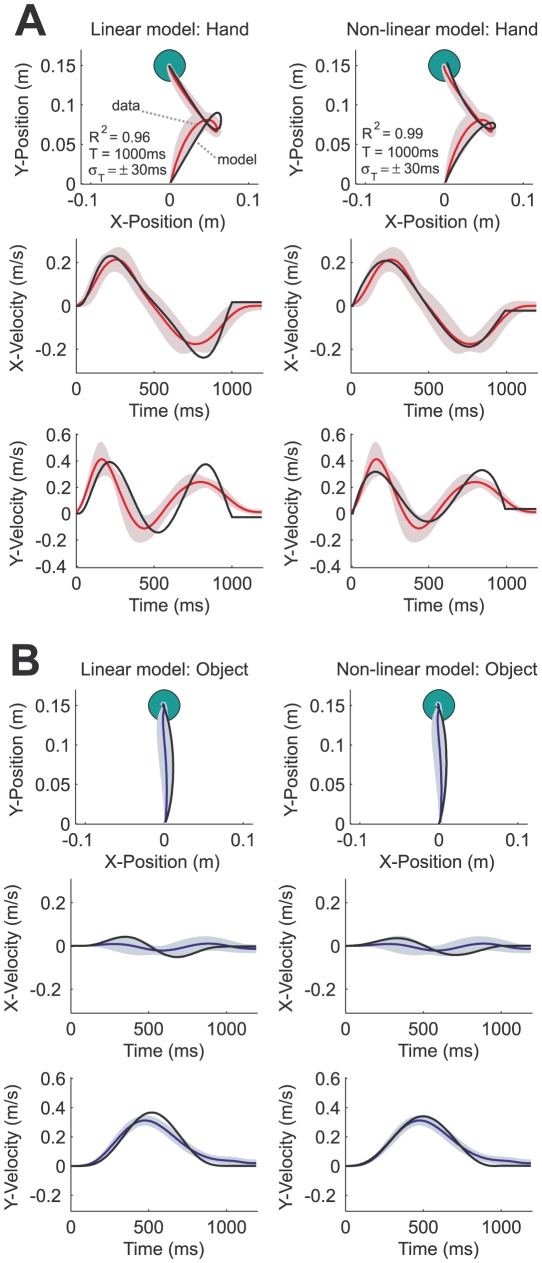
Actual and simulated hand and object paths and velocities for condition B-low. The actual and simulated hand and object paths and velocities for condition B-low in which off-diagonal terms in the viscosity matrix were paired with a low-damped spring. A. The hand path and x- and y-velocities (red lines) with 1 s.e.m. across subjects (standard error ellipse for path plots). The left and right columns show the fits (black lines) of the linear, point-mass optimal control model, and the nonlinear, two-link arm optimal control model, respectively. For the paths 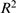 (variance explained by the model), the mean movement duration (which was used in the optimal control simulations) and its standard deviation is shown. B. as in A, but for the object motion (blue lines).

Figure 2 shows the hand (A: red lines) and object (B: blue lines) paths for one of the objects (condition B-low). Here the hand path is complex and deviated substantially from the straight-line movements characteristically observed during free reaching [17],[18]. In this condition the hand path even shows a loop mid-movement. Moreover, rather than the normal bell-shape velocity profiles of reaching movements we observed biphasic and triphasic velocity profiles in the x- and y-direction respectively (Figure 2A). In contrast the motion of the object (Figure 2B) showed a slightly curved movement with a more bell-shaped velocity profile.

**Figure 3 pcbi-1000419-g003:**
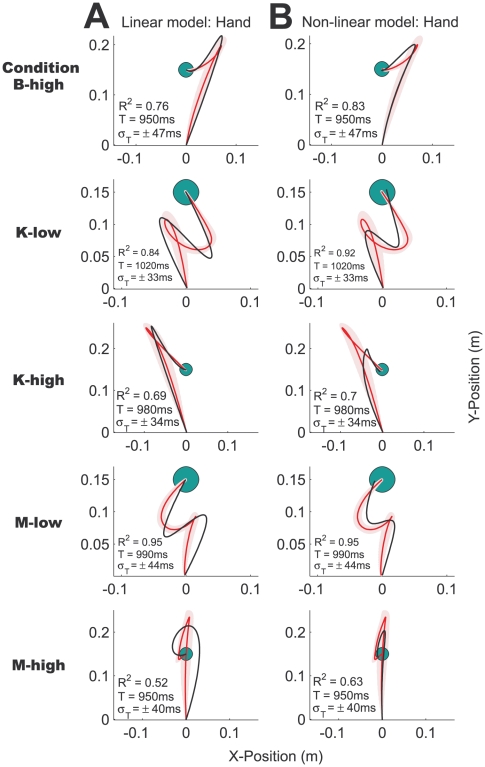
Actual and simulated hand paths. The actual and simulated hand paths for the 5 conditions not shown in Figure 2 (in the same format). A. The fits (black lines) of the linear, point-mass optimal control model, and B. the nonlinear, two-link arm optimal control model, respectively.

**Figure 4:**
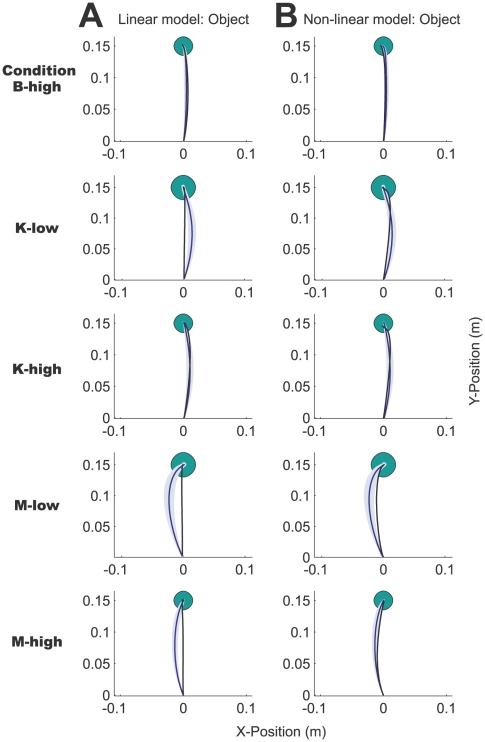
Actual and simulated object paths. The actual and simulated object paths for the 5 conditions not shown in Figure 2 (in the same format). A. The fits (black lines) of the linear, point-mass optimal control model, and B. the nonlinear, two-link arm optimal control model, respectively.

Similar complex hand paths were produced across the other five conditions (Figure 3). A variety of paths were observed: Subjects made S-shaped (condition M-low), mirror S-shaped (condition K-low) and looped paths (condition B-low) in the low damping conditions that required them to decelerate the mass actively. In contrast, the subjects' hands overshot the target in the high damping conditions, veering to the left (condition K-high), to the right (condition B-high) and passing through the target (condition M-high). A way of conceptualizing the high damping conditions is to imagine dragging the object through a very viscous fluid, hence accelerating the mass as much as possible initially becomes crucial for subjects to finish a trial successfully. We also found that in most conditions the object path was not straight but slightly curved (Figure 4). In conditions K-low and K-high, we observed a slight rightward curvature, while in conditions M-low and M-high the object path was substantially curved to the left.

We compared the subjects' performance to the predictions of an optimal feedback controller. The optimal control law can be computed given the dynamics of the system under control and a cost function on the movement. We extended two existing optimal feedback control models (see Methods for details): a Linear-Quadratic-Gaussian controller with signal-dependent noise, in which the hand was modeled as a simple point-mass [5], and a non-linear optimal control model, which included the full dynamics of a two-link arm [10]–[12]. We modified these two optimal control models to include the dynamics of the different mass-spring-damper objects in the state update equations, assuming that by the end of learning the optimal controller had full knowledge of the dynamics of the objects under control. We constructed a cost function with 5 terms: effort and final positional and velocity errors of the hand and object. This cost function has 4 parameters that change the relative weighting of the different cost terms and based on previous studies we constrained the cost to have only two adjustable parameters. These were fit to the data and fixed to be the same for all object conditions (see Methods). Introduction of a sensorimotor delay did not change the results appreciably (see Text S1 and Figure S1).

Figure 2 shows the model fits (black lines) of the linear, point-mass optimal control model (on the left) and the non-linear, two-link arm optimal control model (on the right) for object condition B-low. Both models capture the salient features of the experimental data and show bi- and triphasic velocity profiles for the hand motion, a more bell-shaped velocity profile for the object motion and the loop mid-movement in the hand path. The model explains 96% (linear model) and 99% (non-linear model) of the variance of the experimental data.

Similarly, for the remaining conditions (Figure 3) both optimal control models yield good quantitative fits to the experimental data and overall explain 79±16% (Figure 3A: linear model) and 84±14% (Figure 3B: non-linear model) of the variance across the conditions and subjects. The prominent characteristics of the subjects' behavior such as the S-shaped paths and overshoots are all captured by the two models. The non-linear, two-link arm optimal control model provides slightly better fits to the experimental data than the linear, point-mass optimal control model. Movement in the x-direction is usually less pronounced due to geometrical constraints and higher control costs in the non-linear model. For example, in condition M-high the linear model substantially overestimates movement in the x-direction, whereas the non-linear model captures the overshoot behind the target seen in the subjects' behavior. Features of the subjects' movements such as the curvature in the hand path in condition B-high and the asymmetry of the S-shaped path in condition M-low are also captured by the non-linear optimal control model. Note that the loop in the path in condition K-low is not accounted for by either optimal control model. Three subjects made loop-like movements whereas the others completed the task with the mirrored S-shaped path predicted by both optimal control models (see Discussion).

Both optimal control models fit the experimental object paths well (Figure 4A: linear model, B: non-linear model) with near straight predicted object paths. However, the non-linear model also shows the correct curvature of the object path across all conditions. For example in conditions K-low, M-low and M-high the linear model predicts a near-straight object path, whereas the non-linear model captures the rightward and the leftward curvature of the object paths. Both are the result of less pronounced hand movement in the x-direction in the non-linear optimal control model due to geometrical constraints and higher control costs as discussed above.

Some of the variance of the hand position signal over time is already explained by the fact that the movement starts at the start point and ends at the target. To provide a null-model for the 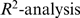 we used non-adaptive versions of the two optimal control models, that is one of the hand alone, moving from the start position to the target in the same time as subjects in the experiment. We computed 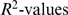 between the optimal control predictions and the experimental data as before (see 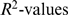 of the non-adaptive controller in Table 2 for the linear and Table 3 for the non-linear model). Note that in principle 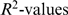 as defined in the Data Analysis (see Methods) can be negative if the predictions are very different from the experimental data. All 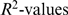 of the non-adaptive controllers are substantially lower than those of the optimal control models including object dynamics. For the linear model 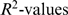 were from 0.41 (Condition M-low) to 0.84 (Condition M-high) lower. For the non-linear model they were from 0.4 (Condition M-low) to 1.03 (Condition M-high) lower. The 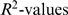 for the low damping conditions are slightly higher than those in the high damping conditions because the movement in the y-direction is fairly similar to the experimental data. The 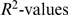 for the high damping conditions are very low as the subjects' movements are very fast in the y-direction and the target is being overshot. In conclusion, the optimal control models with object dynamics included are substantially better at explaining the variance of the experimental data.

**Table 2:** 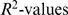
 of the linear optimal control model.

	B-low	B-high	K-low	K-high	M-low	M-high	mean±*σ*
**incl. obj. dyn.**	**0.96**	**0.76**	**0.84**	**0.69**	**0.95**	**0.52**	**0.79±0.16**
**w. mod. unc.**	**0.97**	**0.90**	**0.94**	**0.79**	**0.97**	**0.72**	**0.88±0.17**
**w/o obj. dyn.**	**0.38**	**−0.08**	**0.09**	**−0.07**	**0.53**	**−0.27**	**0.1±0.3**
**Subject 1**	0.92	0.45	0.7	0.74	0.97	0.57	**0.72±0.2**
**Subject 2**	0.91	0.91	0.96	0.93	0.97	0.86	**0.92±0.04**
**Subject 3**	0.93	0.82	0.75	0.63	0.88	0.42	**0.74±0.19**
**Subject 4**	0.91	0.97	0.90	0.99	0.89	0.77	**0.9±0.08**
**Subject 5**	0.98	0.84	0.73	0.8	0.93	0.86	**0.85±0.09**
**Subject 6**	0.83	0.71	0.83	0.81	0.91	0.1	**0.7±0.3**
**mean±** ***σ***	**0.91±0.05**	**0.78±0.18**	**0.81±0.1**	**0.81±0.12**	**0.92±0.04**	**0.59±0.3**	**0.81±0.12**

The table shows 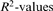 and their means±standard deviations for the linear optimal control model fitted to the mean trajectory across subjects (see Figures 2–4) for the model including the object dynamics (incl. obj. dyn.), with uncertainty about the internal model and incomplete learning (w. mod. unc. - see Figure 6) and using a non-adaptive controller without the object dynamics (w/o obj. dyn.). In addition, 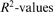 for the linear optimal control model including the object dynamics are shown fitted on an individual subject basis (see Figures S2, S3, S4, S5, S6, and S7).

**Table 3:** 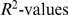
 of the non-linear optimal control model.

	B-low	B-high	K-low	K-high	M-low	M-high	mean±*σ*
**incl. obj. dyn.**	**0.99**	**0.83**	**0.92**	**0.7**	**0.95**	**0.63**	**0.84±0.14**
**w/o obj. dyn.**	**0.47**	**−0.15**	**0.14**	**−0.22**	**0.55**	**−0.4**	**0.07±0.39**
**Subject 1**	0.94	0.6	0.88	0.80	0.93	0.58	**0.79±0.16**
**Subject 2**	0.93	0.97	0.95	0.90	0.96	0.81	**0.92±0.06**
**Subject 3**	0.95	0.88	0.78	0.69	0.89	0.52	**0.79±0.16**
**Subject 4**	0.95	0.98	0.97	0.94	0.91	0.74	**0.91±0.09**
**Subject 5**	0.97	0.94	0.86	0.89	0.92	0.8	**0.89±0.06**
**Subject 6**	0.89	0.75	0.85	0.83	0.94	0.27	**0.75±0.25**
**mean±** ***σ***	**0.94±0.03**	**0.85±0.15**	**0.88±0.07**	**0.84±0.09**	**0.92±0.02**	**0.62±0.2**	**0.84±0.11**

The table shows 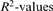 and their means±standard deviations for the non-linear optimal control model fitted to the mean trajectory across subjects (see Figures 2–4) for the model including the object dynamics (incl. obj. dyn.) and using a non-adaptive controller without the object dynamics (w/o obj. dyn.). In addition, 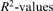 for the non-linear optimal control model including the object dynamics are shown fitted on an individual subject basis (see Figures S2, S3, S4, S5, S6, and S7).

To analyze the performance of individual subjects on the task, the optimal control analysis was repeated on a subject-by-subject basis for both models (see Methods for details). Table 2 shows the 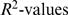 of this new analysis for the linear model and Table 3 for the non-linear model respectively. Figures S2, S3, S4, S5, S6, and S7 show the hand paths overlaid with the optimal control predictions for condition B-low to condition M-high respectively. What becomes apparent from this analysis is that some subjects, on average, performed closer to the optimal control predictions than others (e.g. subject 2 vs subject 6). In addition, although the hand paths of subjects are overall very similar, in some conditions one subject performed slightly differently from the rest (e.g. subject 1 in conditon B-high, subject 3 in condition B-high and subject 1 in condition M-high). It is these conditions that have the lowest 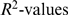 and the individual subject fits slightly improve the model fits for these conditions suggesting that between-subject differences in 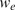 and 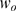 partially account for the lower 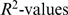. Overall this analysis does not change the main result. The individual subject fits are, on average across conditions, as least as good as those fitted to the mean trajectory (see Tables 2 and 3). Furthermore, the non-linear model still fits the data slightly better than the linear model.

We performed a sensitivity analysis of both optimal control models to the particular weighting used in the cost function. Varying the velocity, effort and object weight over a ten fold range (either smaller or larger) had little effect on the percentage of variance explained (see Text S1 and Figures S8, S9, S10, S11, S12, and S13 for details).

**Figure 5:**
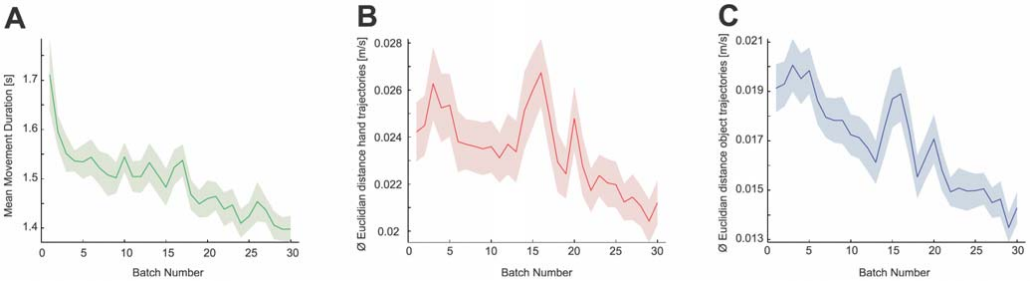
Learning object dynamics. A. Mean movement duration across subjects and conditions as a function of batches of 20 trials. Shaded area shows one s.e.m. across subjects. B. Mean Euclidian distance between subjects' hand trajectories averaged across subjects and conditions. Shaded area shows one s.e.m. across subjects. C. as in B, but for the object trajectory.

To assess learning across the course of the first 600 trials to the 0° target alone (that was the minimum that all subjects were required to perform), we analyzed the movement trial duration averaged across batches of 20 trials (Figure 5A). With time subjects became faster and adapted to the progressively stricter time criterion that was imposed during the experimental sessions. A paired t-test shows a significant difference between the movement duration of the first and the last batch of the experiment 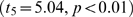.

We also analyzed the way that subjects' trajectories changed throughout learning. In particular we wished to assess whether subjects all showed similar patterns of adaptation at intermediate stages of learning that is whether their trajectories were similar during the learning process. To assess this we developed a measure of the between-subject variability. For each batch of 20 trials we computed the average Euclidian distance between the hand and object trajectories (averaged over the batch) of all pairs of subjects. Low values indicate that all subjects produce similar trajectories and high values represent dissimilar trajectories (see Methods). Subjects started out with very different hand and object trajectories at the beginning of the experiment and their behavior became more similar as they improved their performance (Figure 5B and 5C). A paired t-test between the first and the last batch of the experiment shows a significant difference in the variability measure (hand trajectory: 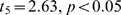; object trajectory: 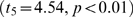 suggesting that the improved performance was due to subjects converging to similar trajectories. Therefore although the final movements were similar across subjects the pattern of trajectory change during learning was idiosyncratic. Analysis of individual conditions (see Figure S14) shows that this is true for all conditions except K-low and K-high. In condition K-low, three subjects made looped movements rather than the inverted S-shape performed by the remaining subjects. The fact that not all subjects converged to the same solution is reflected in the slightly increasing variability measure. Similarly in condition K-high, subject 3 (see Figure S5) performed the task differently from the rest. When subject 3 is removed from the analysis (see Figure S14), convergence of behavior for the remaining subjects is found as before.

**Figure 6:**
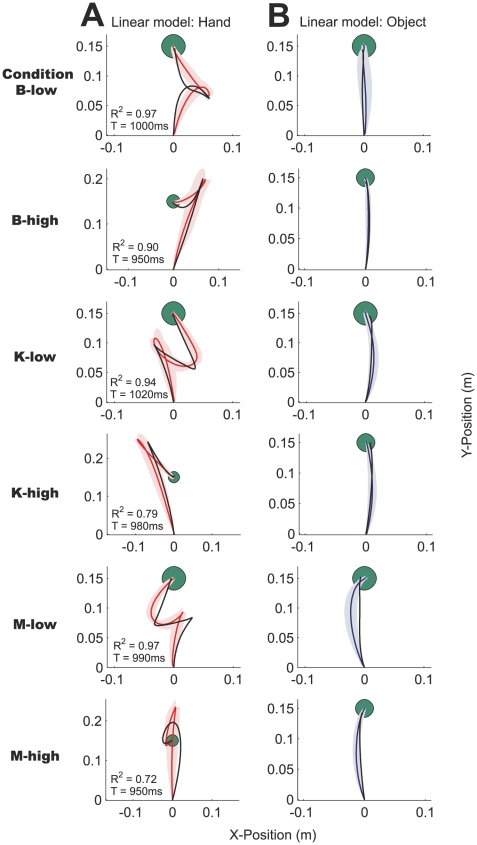
Predictions of the linear optimal control model with model uncertainty and incomplete learning. A. The actual and simulated hand paths for all 6 conditions (in the same format as Figure 2). The fits (black lines) of the linear, point-mass optimal control model with model uncertainty and incomplete learning. B. as in A, but for the object motion (blue lines).

Even after the long exposure that subjects had to the dynamics of the objects during the experiment, they failed to finish the task successfully within the time limit in some trials. Besides the effects of motor noise, another factor that might have contributed is that subjects did not fully learn the objects' dynamics and that a residual uncertainty about the internal model remained. To investigate the effects of model uncertainty and incomplete learning, we adapted the linear optimal control model above in accordance with [19]. In Izawa et al. 2008 incomplete learning was modelled as uncertainty with regard to the internal model. The internal model was represented as 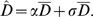. The parameter 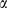 expresses incomplete learning, e.g. 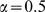 indicates that only 50% of the internal model is learnt. The parameter 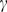 is a Gaussian random variable with standard deviation 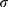 that captures the uncertainty about the internal model. We adapted the linear optimal control model to include model uncertainty (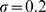) and incomplete learning (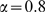), which are the same values used by Izawa et al. (see Text S1 for details). The 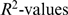 of the simulations are shown in Table 2 and the hand and object path in Figure 6. The model fits are considerably better than for the linear model without model uncertainty (an average 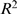 of 88% versus 79%) suggesting that subjects remained to some degree uncertain how to predict the consequences of their actions on the object and that they adjusted their movement strategy accordingly.

We also tested our model on previous experimental data [13] of a relatively simple mass-spring object. The task was to transport a mass-on-a-spring to a target (for details see [13]). Effects of varying movement distance, movement duration and resonant frequency of a simple mass-spring object on human object manipulation were examined. However, in contrast to the current study, there were no off-diagonal terms in the object dynamics. Dingwell et al. used a smoothness criterion on the object path to explain their data. In their study they set out to model the effects of changing the movement distance and movement duration for a fixed resonant frequency of the spring (Experiment B). Our point-mass optimal control model predicts a transition from approximately uniphasic velocity profiles of the hand for slow movements to triphasic velocity profiles for faster movements. In addition, the model predicts that the effect of increasing the movement distance results in scaling the velocity profiles without changing its shape. Both these predictions were observed experimentally (Figure S15 shows experimental data with optimal control model predictions). In an additional experiment Dingwell et al., examined the influence of the resonant frequency of the spring on the velocity profiles of both object and hand (Experiment C). Our point-mass optimal control model yields the same velocity profiles when the resonant frequency is kept constant, independent of how the values of the spring constant and object mass are chosen. High resonant frequencies predict triphasic velocity profiles whereas low resonant frequencies predict uniphasic velocity profiles for the same movement time as the optimal control solution. The optimal control model always yields uniphasic velocity profiles for the object. All these features were observed experimentally (Figure S16 shows experimental data with optimal control model predictions).

## Discussion

Our study suggests that the framework of optimal feedback control in motor neuroscience can be extended to the control of objects with internal degrees of freedom and our results underline the generality of the optimal control framework as a basis of motor coordination. The strength of this theory has been to show that the redundancy inherent in biological motor systems is exploited in a goal directed way and that the variability patterns that emerge are in fact optimal given the noise characteristics of biological systems. Optimal control theory has been able to incorporate competing task goals [8] and explain task-dependent behavior [9] that other theories of motor control such as the desired trajectory hypothesis [20],[21] cannot account for. Previously, optimal control theory has been applied to movements of the arm alone. In this study, we extend the linear point-mass optimal control model by incorporating the dynamical system equations of the different spring-mass-damper systems into the state update equations and by adding end position and velocity of the object to the overall movement cost. We show that complex behavior can be understood with a simple cost function.

Across the six different object conditions, the final hand paths were quite dissimilar and all deviated substantially from the near straight hand paths seen during normal reaching movements. The object path shows a slight curvature in most conditions. Our adapted version of the simple point-mass optimal control model provided a good fit to the data explaining 79±16% of the variance across all cases. However, there were some notable failures of the simple model: for example it often overestimated the hand movement in the x-direction (Figure 3) and also did not show the right curvature of the object path (Figure 4). In most optimal control models in the literature the dynamics of the arm are neglected and the hand is simply modeled as a point mass [5],[8],[9],[19]. Numerous phenomena of human movements have been explained using this simple model [5]–[7] and often it has not been necessary to include the full dynamics of the arm. Rather than having been a matter of deliberate model choice, the Linear-Quadratic-Gaussian (LQG) case is the only one for which there exists a closed-form solution. In contrast, iterative LQG algorithms can find approximate solutions to non-linear optimal control problems such as those including realistic dynamics of the arm [10]–[12]. We built on this previous work and in addition included the dynamics of the different spring-mass-damper systems. In this study, in five out of six cases the two-joint arm model predicted the data better than the simple point-mass model for the same number of free parameters. In addition, it predicts the curved object paths seen in the experimental data, some of which are not predicted by the simply point mass model (Figure 4) and none of which could be predicted by a smoothness criterion on the object trajectory. Our results lend weight to using such models for experiments where geometry and dynamics of the arm matter. In the future, it will be interesting to apply the optimal control framework to increasingly more complex situations and also attempt to include facets of human movements that are traditionally not captured by optimal control models such as impedance control.

We also set out to model behavioral data from a previous experiment [13], in which subjects moved simple two-dimensional mass-spring objects whose dynamics were intuitive and possibly known to subjects by experience. The velocity profiles of the hand differed substantially from the classic bell-shaped velocity profiles observed during normal reaching movements and in some instances the velocity profiles were triphasic. Previously, Dingwell et al. modeled the data by applying a smoothness criterion to the object trajectory rather than the hand trajectory as in past models of trajectory planning [20],[21]. In this case, for mathematical reasons, smoothness was defined as minimum-crackle (i.e. the fifth derivative of position) rather than minimum-jerk (i.e. the third derivative of position) as in previous studies. The model provided good fits to the experimental data and made reliable predictions regarding the shape and scaling of the velocity profiles and their dependence on movement time, movement distance and resonance frequency of the mass-spring object. Our adapted optimal control model is also able to predict the same data by simply specifying as task requirements that both hand and object be moved to the target and come to a complete stop in a certain time and that the effort of the movement be minimized. The model makes no assumptions about the particular shape of either the hand or the object trajectory and it provides a normative explanation of the subjects' behavior. The complex hand velocity profiles and the nearly smooth object trajectory are simply a result of an effort-accuracy trade-off given the dynamics of the different systems to be controlled.

Although both optimal control models provide very good quantitative fits to the data, they do not account for some aspects of the behavioral results. For example, in condition K-low three subjects chose to make looped movements rather than the inverted S-shape predicted by the optimal control model and performed by the remaining subjects (see Figure S4). First, in the model we assume identical dynamics and kinematics for all subjects whereas clearly subjects have arms with different properties and variation in the dynamics will lead to some variation in the optimal trajectories. Second, a key strength of our model is that all the six object conditions were fit using the same cost function parameter settings. This assumes that the weighting of effort efficiency and the importance of the object remains the same and that these two terms are traded-off in the same manner across subjects and conditions. In accordance with this explanation, we find that fitting on an individual subject basis slightly improved the average variance explained slightly for the linear optimal control model. In the future, it will be interesting to investigate the task-dependency of these parameters when object dynamics and task goals are varied.

Another observation is that although subjects had extensive training manipulating the objects, they were not successful at finishing the task in the time limit on every trial. Our optimal control models assume complete knowledge of the system dynamics and that the subjects' learning process was complete at the end of the experiment. A recent paper modeled uncertainty about the internal model of the system under control in a force-field experiment [19] and using their approach we show, that the subjects' behavior can be better accounted for when taking model uncertainty into account. This suggests that subjects were to some degree uncertain of how to predict the consequences of their actions on the object and that they adjusted their movement strategy accordingly. Initially the object dynamics of the mass-spring objects were completely unknown to the subjects and the task required subjects to learn a new mapping between their motor commands and their consequences, i.e to acquire an internal model of the object's dynamics [3],[4]. Note that in all optimal control models described above, the system's dynamics were incorporated into the model by including the dynamical system equations of the different spring-mass-damper objects into the state update equations. Hence, the current study does not attempt to model learning in an optimal control framework but rather looks at the end point of learning assuming that the dynamics of the system are already known. Recently, studies have started to shed light on adaptation in an optimal control framework [22] and in the future, it will be interesting to investigate the adaptation processes that occur during learning of novel object dynamics.

## Methods

### Experimental Setup

After providing written informed consent 6 right-handed subjects (3 male, 3 female, age 19–28) participated in the study. The experimental protocol was approved by a local ethics committee. Subjects were naive to the purpose of the experiment and none of the subjects reported any sensory or motor deficits. While seated, subjects used their right hand to grasp the handle of a vBOT force-generating robotic manipulandum, which could be moved in the horizontal plane (for details, see [23]). The position and velocity of both hand and the virtual object were computed online at 1000 Hz. Subjects could not see their arm but the positions of the object and hand were displayed in the plane of the arm using a reflected rear-projection system. The position of the object was displayed as a circular disk (yellow; 1 cm radius) which was connected by a yellow virtual rubber band to the position of the hand, also represented as a circular disk (blue; 0.5 cm radius). The vBOT could apply forces to the hand and this was used to simulate objects with different mass-spring-damper properties.

### Simulation of Object Dynamics

As a prototypical object with internal degrees of freedom in two dimensions we simulated a damped point mass, attached to the hand by a spring with an equilibrium position identical to the position of the hand. Let 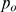 be the position of the object, 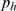 be the position of the hand, 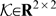 be a spring constant matrix, 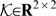 define a damping (viscosity) matrix for the system and 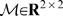 be the mass matrix of the object (all these parameters are specified in Cartesian coordinates). The differential equation describing the motion of the object can be written as:
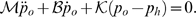



We also applied complex forces to the hand that depended on both the position of the hand and position and velocity of the object (the combination is similar to a spring which has stiff hinges at each end so that the damping on the object plays through to the hand):
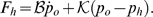



The matrices used for the standard two-dimensional mass-spring-damper were:
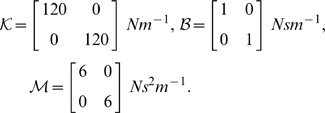



However, the dynamics of such a standard spring are relatively simple and most probably known to the subjects. We were interested in the control of objects with complex dynamics for which subjects are unlikely to have had prior experience and for which there would not be an intuitive way of controlling them. Hence, we created six different complex dynamic objects by introducing x-y dependencies for the spring constant, viscosity and mass matrices. These three conditions were paired with low and with high damping (i.e. diagonal terms in the viscosity matrix). In conditions B-low & B-high, we included off-diagonal terms for the viscosity matrix. This is similar to a velocity-dependent curl field that is often used in studies of dynamics learning [14]–[16], except the field is applied to the object mass rather than the hand. We only specify the parameters that were changed from the standard diagonal mass-spring-damper system:
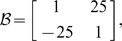
(Condition B-low)

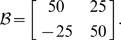
(Condition B-high)


In conditions K-low & K-high we introduced off-diagonal terms in the spring constant matrix. This can be conceptualized as introducing an x-y dependency, in which a movement along one axis will result in a restoring force in both x- and y-direction:
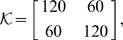
(Condition K-low)

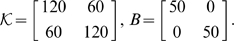
(Condition K-high)


In conditions M-low & M-high, we introduced off-diagonal terms in the mass matrix of the object. This can be thought of as an acceleration along one axis simultaneously resulting in acceleration along the orthogonal axis:
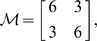
(Condition M-low)

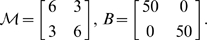
(Condition M-high)


Overall the simulation of these dynamics provided a complex, non-intuitive, yet learnable environment that is highly unlikely to have been experienced in the real world. Each of the six conditions was performed in a block of trials and subjects performed the different blocks on consecutive days. The order of the six blocks was counterbalanced as much as possible to avoid systematic training effects and biases. Subjects performed condition B, K and M in a different order (that is, subjects 1 & 4 started with condition B, subject 2 & 5 with condition K and subject 3 & 6 with condition M). In addition, half of the subjects (subject 1, 2 & 3) always started with a low-damped spring (“low”) for a given condition, whereas the other half (subject 4, 5 & 6) always started with a high-damped spring (“high”) (see Table S1 for details).

### Experimental Protocol

Subjects started with both hand and object aligned in the starting position and were required to move both the hand and object to the target position (see Figure 1). The target was always 15 cm from the start position and in a direction of either −22.5°, 0° or +22.5° (first training session) or only 0° (second training session and test sessions) from straight ahead. Based on a pilot experiment we varied the target size and speed criteria so that the task difficulty (number of sessions to reach the 25% correct trials criterion) was roughly similar across objects (see Table 1). Thus, the radius of the target was 2 cm for low damping conditions B-low, K-low and M-low and 1.5 cm for the high damping conditions B-high, K-high and M-high respectively. To expose subjects to the full dynamics of the object and to encourage exploration, subjects completed a training session of 180 trials with three different training directions (60 trials in each direction). One of the three targets was displayed at random and the target had to be reached within 1.5±0.2 s. To succeed in the task, the hand and object both had to be within the target with a speed below 0.1 ms^−1^ (low damping) and 0.02 ms^−1^ (high damping) within the duration limits. Feedback about success was given after every trial. Every successful trial was rewarded by a point and for unsuccessful trials the time above criterion was displayed. The first training session was followed by a second training session in which only the 0° target was presented and the time window was reduced to 1±0.2 s over the course of 400 trials. Finally, subjects completed the actual test session of 200 trials to the 0° target, which was repeated until they reached 25% correct trials.

### Optimal Feedback Control Models

#### Linear control model (point mass)

In the first instance, we used an optimal control model based on [5] and included the dynamics of the different mass-spring-damper systems. The hand was modeled as a 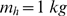 point mass, controlled by a pair of orthogonal actuators along the x- and y-axes of the horizontal plane. The two actuators were modeled as second order linear muscle filters, with time constants 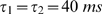.

Let 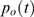, 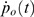, 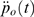, 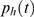, 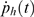, 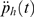 be the two-dimensional position, velocity, and acceleration of the object and hand respectively, 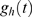, 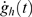, 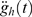 be the second-order linear muscle filters and its derivatives, and 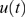 be the control signal. The time variable runs from 0 to the final time 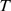, which was chosen in accordance with the experimental data (see Data Analysis). For simulations in discrete time we used a time step of 10 ms. The differential equations describing the dynamical system were as follows:
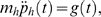


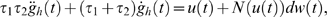


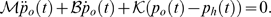



The control signal in the human motor system is contaminated by signal-dependent noise [24]–[26], which was implemented using 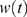 corresponding to Gaussian noise variable and 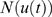 corresponding to control-multiplicative noise as follows:
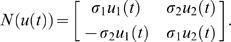



The noise term 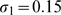 corresponds to noise in the same direction as the control signal, whereas 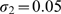 corresponds to noise in the direction orthogonal to the control signal.

The target location, which is the same for both object and hand, is denoted by 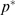 and hence the state of the system can be fully captured by a fourteen-dimensional state vector:
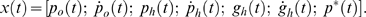



In accordance with previous optimal control models, we used a mixed cost function with weights defining the relative importance of the different cost terms and added position and velocity requirements for the object:
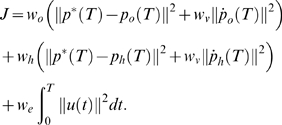



The five cost terms correspond to positional accuracy and stopping at the target of both object and hand, respectively, and to effort. 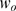 and 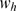 determine the relative importance of object and hand. In the simulations, 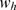 was set to 1 and 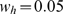 was used as an overall fit to the data. The weight 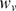 determines the importance of coming to a complete stop relative to reaching the target and 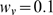 was used as in previous studies [5]. The weighting for effort is a free parameter and 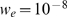 was used for all conditions as an overall fit to the data.

Note that values for 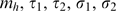 were chosen to be compatible with the biomechanics of the arm and were not fit to the data. They are the same as in previous optimal control models [5].

#### Nonlinear control model (2-joint arm)

We used an adapted version of an iterative LQG method for locally-optimal feedback control, which can solve optimal control problems for non-linear systems [10],[11],[12]. We adapted the Matlab implementation of the algorithm that is available at www.cogsci.ucsd.edu/~todorov. The arm was modeled as a two-joint arm, including the shoulder and the elbow.

Let 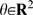 be the joint angle vector (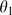: shoulder angle, 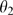: elbow angle), 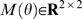 the positive definite inertia matrix, 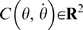 a vector representing centripetal and Coriolis forces, 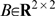 the joint friction matrix, and 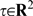 the joint torque. As before, 10 ms was used as the discrete time step. The differential equations describing the dynamical system were as follows:
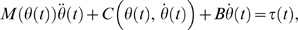


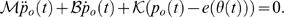
where 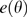 is the forward kinematic transformation of joint angles to the position of the arm's endpoint in Cartesian coordinates. The joint angles at the beginning of the movement were 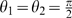. The same parameters for the arm model were used as in [11].

The eight-dimensional state vector can be written as 
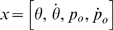
 and the target position in joint coordinates is denoted by 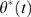 and in Cartesian coordinates by 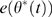 respectively.

Similar to the first model, we used a mixed cost function and added position and velocity requirements for the object:
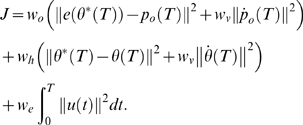



As before, the five cost terms correspond to positional accuracy and stopping at the target for both object and hand, respectively and to effort. 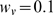 and 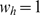 was used as in the previous model. 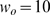 and 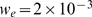 was used as an overall fit to the data.

For more details on the two optimal control models, see Text S1.

### Data Analysis

The last 25 successful trials for each condition and subject were analyzed (that is a total of 150 trials for every condition). The start of a trial was defined as the subject crossing a speed threshold of 0.01 ms^−1^ and the data was aligned accordingly. Data was recorded for at least 1.2 s, which was the slowest permissible correct trial. The velocity data was filtered using a fifth order low-pass Butterworth filter with a cut-off frequency of 8 Hz. For every condition, positional and velocity data of the 150 trials (i.e. the last correct 25 trials of each subject) were averaged and resampled at 100 Hz. This is acceptable as the movement durations of the 150 trials were very similar (see Figure 2 and 3). The final movement time 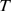 of the mean trajectory was defined as the moment when all four task criteria were fulfilled (i.e. when both hand and object were within the target region with a speed below 0.1 ms^−1^ (low damping conditions) and 0.02 ms^−1^ (high damping conditions)) and used in the optimal control simulations. The mean start and end positions of the hand from the experiment were used in the optimal control simulations. The linear optimal control simulation was run 150 times and the average trajectory was computed. The non-linear optimal control simulation is not stochastic and hence was run only once. The mean hand trajectory (averaged across subjects and trials) was calculated and 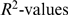 for the average position signal in x and y over time were computed for both optimal control simulations (note that this variance measure does neither assess subject-by-subject nor trial-by-trial variance):
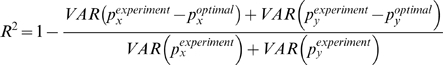



To provide a null-model for the 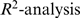, we computed optimal trajectories and 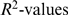 for the non-adaptive versions of the two optimal control models. For this, the object dynamics were removed from the state update equation as were position and velocity terms for the object in the cost function. The same parameter settings for the optimal control simulations were used (i.e. T, noise characteristics etc.) and 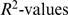 between the optimal control predictions and the experimental data for each condition was computed as before.

To analyze the performance of individual subjects, optimal control simulations and 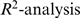 was repeated for the last 25 correct trials of every subject. The same parameters were used as before except 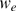 and 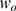 were now fit for each subject individually rather than across subjects (see Table S2 for fitted values).

Learning for reaches to the 0° target was analyzed in the second training sessions and the first test session that all subjects completed. The first training session in which subjects moved to three different target directions was excluded from the analysis to rule out potentially confounding aftereffects from previous conditions and increased variability due to movement to different target directions. Additional test sessions that some subjects required to reach the performance criterion (Table 1) were not included in the analysis for comparability reasons. The 600 trials were grouped into batches of 20 trials resulting in a total of 30 batches.

Movement duration was defined as the time elapsed between exceeding a speed threshold of 0.01 ms^−1^ at the beginning of the trial until reaching a predefined speed threshold in the target area for both hand and object (see Experimental Protocol). The data was averaged across conditions and across subjects and comparisons between the first and the last batch were made using a paired t-test.

To assess variability between subjects across the course of the experiment, we computed the average Euclidian distance between hand trajectories and between object trajectories. The start of a trial was defined as the subject crossing a speed threshold of 0.01 ms^−1^ and the data was aligned accordingly. To allow comparisons between movements of different durations, we only considered the first 1.2 s of the movement (as data was recorded for at least 1.2 s). For every batch of 20 trials an average trajectory was computed. The average Euclidian distance between a given subject's average trajectory and the average trajectory of every other subject was calculated resulting in 5 comparisons for every subject. The mean value across the 5 comparisons for every subject and across the 6 conditions for every batch was taken, resulting in one value per batch for every subject, and comparisons between the first and the last batch were made using a paired t-test.

To test the point-mass optimal control model on the results of [13], we modeled the experimental data of a typical subject from Experiment B and Experiment C of the original study. For the final movement time 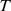 the same value from the fits in the original paper was used. The weighting factor for effort was set to 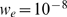, the same as for the previous experiment. The relative importance of hand to object was not fit to the data and simply set to 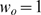.

## Supporting Information

Figure S1 Predictions of the linear optimal control model with incomplete state observation and sensorimotor delay. A. The actual and simulated hand paths for all 6 conditions (in the same format as Figure 2 of the main article). The fits (black lines) of the linear, point-mass optimal control model with incomplete state observation and a sensorimotor delay of 100 ms. B. as in A, but for the object motion (blue lines).
(4.67 MB EPS)

Figure S2 Condition B-low: Actual and simulated hand paths for individual subjects.The actual and simulated hand paths for Condition B-low of all 6 subjects (in the same format as Figure 2 of the main article). A. The fits (black lines) of the linear, point-mass optimal control model, and B. the nonlinear, two-link arm optimal control model, respectively.
(3.86 MB EPS)

Figure S3 Condition B-high: Actual and simulated hand paths for individual subjects. The actual and simulated hand paths for Condition B-high of all 6 subjects (in the same format as Figure 2 of the main article). A. The fits (black lines) of the linear, point-mass optimal control model, and B. the nonlinear, two-link arm optimal control model, respectively.
(3.45 MB EPS)

Figure S4 Condition K-low: Actual and simulated hand paths for individual subjects.The actual and simulated hand paths for Condition K-low of all 6 subjects (in the same format as Figure 2 of the main article). A. The fits (black lines) of the linear, point-mass optimal control model, and B. the nonlinear, two-link arm optimal control model, respectively.
(3.84 MB EPS)

Figure S5 Condition K-high: Actual and simulated hand paths for individual subjects. The actual and simulated hand paths for Condition K-high of all 6 subjects (in the same format as Figure 2 of the main article). A. The fits (black lines) of the linear, point-mass optimal control model, and B. the nonlinear, two-link arm optimal control model, respectively.
(3.41 MB EPS)

Figure S6 Condition M-low: Actual and simulated hand paths for individual subjects. The actual and simulated hand paths for Condition M-low of all 6 subjects (in the same format as Figure 2 of the main article). A. The fits (black lines) of the linear, point-mass optimal control model, and B. the nonlinear, two-link arm optimal control model, respectively.
(3.88 MB EPS)

Figure S7 Condition M-high: Actual and simulated hand paths for individual subjects. The actual and simulated hand paths for Condition M-high of all 6 subjects (in the same format as Figure 2 of the main article). A. The fits (black lines) of the linear, point-mass optimal control model, and B. the nonlinear, two-link arm optimal control model, respectively.
(3.37 MB EPS)

Figure S8 Sensitivity analysis of condition B-low. The plots depict how the goodness of fit changes when *w_v_* and the two fitted parameters *w_o_* and *w_e_* are varied from one tenth to ten times of their fitted values. The values in between were sampled uniformly on a logarithmic scale. A. Linear, point-mass optimal control model. B. Non-linear, two-link arm optimal control model.
(3.89 MB EPS)

Figure S9 Robustness to changes in *w_e_*. Effect of *w_e_* on the hand paths for A. the linear model and B. the non-linear model.
(4.57 MB EPS)

Figure S10 Robustness to changes in *w_o_* (linear model). Effect of *w_o_* on A. hand paths and B. object paths predicted by the linear model.
(4.38 MB EPS)

Figure S11 Robustness to changes in *w_o_* (non-linear model). Effect of *w_o_* on A. hand paths and B. object paths predicted by the non-linear model.
(4.40 MB EPS)

Figure S12 Robustness to changes in *w_v_* (linear model). Effect of *w_v_* on A. hand paths and B. object paths predicted by the linear model.
(1.74 MB EPS)

Figure S13 Robustness to changes in *w_v_* (non-linear model). Effect of *w_v_* on A. hand paths and B. object paths predicted by the non-linear model.
(1.77 MB EPS)

Figure S14 Between-subject variability for individual conditions. A. The average Euclidian distance of the hand trajectory (red lines) for all 6 conditions with 1 s.e.m. across subjects. For condition K-high, we also show the average Euclidian distance for only 5 subjects (black line) excluding subject 3 that performed this condition differently (see Figure S5). B. as in A, but for the object trajectory (blue lines).
(1.30 MB EPS)

Figure S15 Optimal control simulations for Experiment B of Dingwell et al. Hand-velocity profiles for each of the four test conditions of Experiment B for a typical subject. The linear point-mass optimal control model predicts that increasing movement distance simply scales the velocity profile as is observed experimentally. For faster movements a change from a uniphasic to a triphasic velocity profile is predicted and seen in the behavioural data. Thin grey lines correspond to individual trials, thick grey lines represent the average of the thin grey lines, and black lines denote the optimal control predictions of the linear, point-mass model. Similar results were obtained for 13 of 14 subjects. (Figure S15 was adapted with permission from Figure 5 from Jonathan B. Dingwell, Christopher D. Mah, and Ferdinando A. Mussa-Ivaldi. Experimentally Confirmed Mathematical Model for Human Control of a Non-Rigid Object. J Neurophysiol 91(3): 1158-1170, 2004 (J704-3))
(1.32 MB EPS)

Figure S16 Optimal control simulations for Experiment C of Dingwell et al. Hand and object velocity profiles of Experiment C for a typical subject manipulating three different objects (k - object spring constant, m - object mass, f - resonant frequency). The resonant frequency, independent of the particular set of spring constant and object mass, determines the shape of the velocity profile predicted by the linear, optimal control model. For both object #1 (k = 60 Nm^−1^; m = 1.5 kg; f = 1.0 Hz) and object #3 (k = 180 Nm^−1^; m = 4.5 kg; f = 1.0 Hz), which both have the same resonant frequency, the model predicts a triphasic hand velocity profile also observed experimentally. Note that differences in the shape of the velocity profile result from differences in the movement duration. For object #2 (k = 180 Nm^−1^; m = 1.5 kg; f = 1.7 Hz) with a greater resonant frequency the model predicts a uniphasic hand velocity profile also seen in the behavioural data. Thin grey lines correspond to individual trials, thick grey lines represent the average across trials, and black lines denote the optimal control predictions of the linear, point-mass model. (Figure S16 was adapted with permission from Figure 6 from Jonathan B. Dingwell, Christopher D. Mah, and Ferdinando A. Mussa-Ivaldi. Experimentally Confirmed Mathematical Model for Human Control of a Non-Rigid Object. J Neurophysiol 91(3): 1158-1170, 2004 (J704-3))
(1.41 MB EPS)

Table S1 Order of presentation of conditions. The order of presentation of the different conditions was counterbalanced as much as possible between subjects to avoid systematic biases. None of the conditions appears twice in one column. Half of the subjects always started with a low-damped spring for a given condition (“low”) whereas the other half always started with a high-damped spring (“high”).
(0.02 MB PDF)

Table S2 *w_e_*- and *w_o_*-values used for the optimal control simulations fitted to individual subject trajectories.
(0.02 MB PDF)

Text S1 Specifications of state update equations used in the two optimal control models, the LQR with incomplete state observation and sensorimotor delay, the sensitivity analysis and the LQR with model uncertainty and incomplete learning.
(0.07 MB PDF)
