# Supplementary material for: Optimal Control Predicts Human Performance on Objects with Internal Degrees of Freedom
Source: PLoS Comput Biol. 2009 Jun 26;5(6):e1000419. doi: 10.1371/journal.pcbi.1000419 (PMC2694986; doi:10.1371/journal.pcbi.1000419)
Supplement: Table S1 — Order of presentation of conditions. The order of presentation of the different conditions was counterbalanced as much as possible between subjects to avoid systematic biases. None of the conditions appears twice in one column. Half of the subjects always started with a low-damped spring for a given condition (“low”) whereas the other half always started with a high-damped spring (“high”). (0.02 MB PDF) [file pcbi.1000419.s017.pdf]

	1.	2.	3.	4.	5.	6.
**Subject 1 (m)**	B-low	B-high	K-low	K-high	M-low	M-high
**Subject 2 (f)**	K-low	K-high	M-low	M-high	B-low	B-high
**Subject 3 (m)**	M-low	M-high	B-low	B-high	K-low	K-high
**Subject 4 (f)**	B-high	B-low	K-high	K-low	M-high	M-low
**Subject 5 (m)**	K-high	K-low	M-high	M-low	B-high	B-low
**Subject 6 (f)**	M-high	M-low	B-high	B-low	K-high	K-low

Table S1. Order of presentation of conditions. The order of presentation of the different conditions was counterbalanced as much as possible between subjects to avoid systematic biases. None of the conditions appears twice in one column. Half of the subjects always started with a low-damped spring for a given condition ("low") whereas the other half always started with a high-damped spring ("high").
