# Supplementary material for: Optimal Control Predicts Human Performance on Objects with Internal Degrees of Freedom
Source: PLoS Comput Biol. 2009 Jun 26;5(6):e1000419. doi: 10.1371/journal.pcbi.1000419 (PMC2694986; doi:10.1371/journal.pcbi.1000419)
Supplement: Table S2 — we- and wo-values used for the optimal control simulations fitted to individual subject trajectories. (0.02 MB PDF) [file pcbi.1000419.s018.pdf]

	**Linear model**		**Non-linear model**	
	w_e	w_o	w_e	w_o
**Subject 1**	5×10^{-8}	0.05	10^{-2}	10
**Subject 2**	10^{-9}	0.01	10^{-3}	10
**Subject 3**	10^{-7}	0.1	10^{-2}	10
**Subject 4**	10^{-8}	0.05	2×10^{-3}	50
**Subject 5**	5×10^{-9}	0.05	10^{-2}	10
**Subject 6**	10^{-7}	0.1	10^{-2}	50

Table S2. w_e**- and** w_o**-values used for the optimal control simulations fitted to individual subject trajectories**
