# Supplementary material for: Optimal Control Predicts Human Performance on Objects with Internal Degrees of Freedom
Source: PLoS Comput Biol. 2009 Jun 26;5(6):e1000419. doi: 10.1371/journal.pcbi.1000419 (PMC2694986; doi:10.1371/journal.pcbi.1000419)
Supplement: Text S1 — Specifications of state update equations used in the two optimal control models, the LQR with incomplete state observation and sensorimotor delay, the sensitivity analysis and the LQR with model uncertainty and incomplete learning. (0.07 MB PDF) [file pcbi.1000419.s019.pdf]

Supplementary Material - Optimal Control Predicts Human Performance on Objects with Internal Degrees of Freedom

Arne J. Nagengast[1,2,*], Daniel A. Braun[1,3], Daniel M. Wolpert[1]
1 Computational and Biological Learning Lab, Department of Engineering, University of Cambridge, Cambridge CB2 1PZ, United Kingdom.
2 Department of Experimental Psychology, University of Cambridge, Cambridge CB2 3EB, United Kingdom.
3 Bernstein Center for Computational Neuroscience, Albert-Ludwigs Universität Freiburg, 79104 Freiburg, Germany.
$\ast$ E-mail: an261@cam.ac.uk

Optimal Control Models

Here, we provide details about the state update equations used in the two optimal control models. Let $\mathcal{K}$ be the spring constant matrix, $\mathcal{B}$ be the viscosity matrix and $\mathcal{M}$ be the mass matrix of the mass-spring-damper:

$$\mathcal{K} = \begin{bmatrix} k_{11} & k_{12} \\ k_{21} & k_{22} \end{bmatrix}, \qquad \mathcal{B} = \begin{bmatrix} b_{11} & b_{12} \\ b_{21} & b_{22} \end{bmatrix}, \qquad \mathcal{M} = \begin{bmatrix} m_{11} & m_{12} \\ m_{21} & m_{22} \end{bmatrix}.$$

Linear, point-mass optimal control model

Let m_h be the mass of the hand, τ_1 and τ_2 be the time constants of the second order linear muscle filter, which then yields the state space equation:

$$\dot{\vec{x}}(t) = A\vec{x}(t) + B\vec{u}(t)$$

with the matrices

$$A = \begin{bmatrix}
\cdot & 1 & \cdot & \cdot & \cdot & \cdot & \cdot & \cdot & \cdot & \cdot & \cdot & \cdot & \cdot & \cdot \\
\mathcal{A}_{k_x} & \mathcal{A}_{b_x} & -\mathcal{A}_{k_x} & \cdot & \cdot & \cdot & \cdot & \mathcal{A}_{k_{xy}} & \mathcal{A}_{b_{xy}} & -\mathcal{A}_{k_{xy}} & \cdot & \cdot & \cdot & \cdot \\
\cdot & \cdot & \cdot & 1 & \cdot & \cdot & \cdot & \cdot & \cdot & \cdot & \cdot & \cdot & \cdot & \cdot \\
\cdot & \cdot & \cdot & \cdot & \frac{1}{m_h} & \cdot & \cdot & \cdot & \cdot & \cdot & \cdot & \cdot & \cdot & \cdot \\
\cdot & \cdot & \cdot & \cdot & -\frac{1}{\tau_2} & \frac{1}{\tau_2} & \cdot & \cdot & \cdot & \cdot & \cdot & \cdot & \cdot & \cdot \\
\cdot & \cdot & \cdot & \cdot & \cdot & -\frac{1}{\tau_1} & \cdot & \cdot & \cdot & \cdot & \cdot & \cdot & \cdot & \cdot \\
\cdot & \cdot & \cdot & \cdot & \cdot & \cdot & 1 & \cdot & \cdot & \cdot & \cdot & \cdot & \cdot & \cdot \\
\cdot & \cdot & \cdot & \cdot & \cdot & \cdot & \cdot & \cdot & 1 & \cdot & \cdot & \cdot & \cdot & \cdot \\
\mathcal{A}_{k_{yx}} & \mathcal{A}_{b_{yx}} & -\mathcal{A}_{k_{yx}} & \cdot & \cdot & \cdot & \cdot & \mathcal{A}_{k_y} & \mathcal{A}_{b_y} & -\mathcal{A}_{k_y} & \cdot & \cdot & \cdot & \cdot \\
\cdot & \cdot & \cdot & \cdot & \cdot & \cdot & \cdot & \cdot & \cdot & \cdot & 1 & \cdot & \cdot & \cdot \\
\cdot & \cdot & \cdot & \cdot & \cdot & \cdot & \cdot & \cdot & \cdot & \cdot & \cdot & \frac{1}{m_h} & \cdot & \cdot \\
\cdot & \cdot & \cdot & \cdot & \cdot & \cdot & \cdot & \cdot & \cdot & \cdot & \cdot & -\frac{1}{\tau_2} & \frac{1}{\tau_2} & \cdot \\
\cdot & \cdot & \cdot & \cdot & \cdot & \cdot & \cdot & \cdot & \cdot & \cdot & \cdot & \cdot & -\frac{1}{\tau_1} & \cdot \\
\cdot & \cdot & \cdot & \cdot & \cdot & \cdot & \cdot & \cdot & \cdot & \cdot & \cdot & \cdot & \cdot & 1
\end{bmatrix},$$

$$B = \begin{bmatrix}
\cdot & \cdot & \cdot & \cdot & \cdot & \frac{1}{\tau_1} & \cdot & \cdot & \cdot & \cdot & \cdot & \cdot & \cdot & \cdot \\
\cdot & \cdot & \cdot & \cdot & \cdot & \cdot & \cdot & \cdot & \cdot & \cdot & \cdot & \cdot & \frac{1}{\tau_1} & \cdot
\end{bmatrix}',$$

where the variables in the A matrix correspond to:

$\mathcal{A}_{k_x} = -\frac{m_{22}\times k_{11} - m_{12}\times k_{21}}{|\mathcal{M}|}$ $\mathcal{A}_{b_x} = -\frac{m_{22}\times b_{12} - m_{12}\times b_{22}}{|\mathcal{M}|}$

$\mathcal{A}_{k_{xy}} = -\frac{m_{22}\times k_{12} - m_{12}\times k_{22}}{|\mathcal{M}|}$ $\mathcal{A}_{b_{xy}} = -\frac{m_{22}\times b_{12} - m_{12}\times b_{22}}{|\mathcal{M}|}$

$\mathcal{A}_{k_{yx}} = -\frac{m_{11}\times k_{21} - m_{21}\times k_{11}}{|\mathcal{M}|}$ $\mathcal{A}_{b_{yx}} = -\frac{m_{11}\times b_{21} - m_{21}\times b_{11}}{|\mathcal{M}|}$

$\mathcal{A}_{k_y} = -\frac{m_{11}\times k_{22} - m_{21}\times k_{12}}{|\mathcal{M}|}$ $\mathcal{A}_{b_y} = -\frac{m_{11}\times b_{22} - m_{21}\times b_{12}}{|\mathcal{M}|}$.

For computational reasons the problem needs to be discretized and the discretization was performed using a matrix exponential with time step $t = 0.01s$.

Non-linear, two-link arm optimal control model

The algorithm developed by Todorov & Li (2005) [1] was used, which is available from http://www.cs.washington.edu/homes/todorov/, was used. Their dynamics model of the arm was left unchanged and the following state update matrix for the object was added to the existing code:

$$A = \begin{bmatrix} 0 & 1 & 0 & 0 & 0 & 0 \\ \mathcal{A}_{k_x} & \mathcal{A}_{b_x} & -\mathcal{A}_{k_x} & \mathcal{A}_{k_{xy}} & \mathcal{A}_{b_{xy}} & -\mathcal{A}_{k_{xy}} \\ 0 & 0 & 0 & 0 & 0 & 0 \\ 0 & 0 & 0 & 0 & 1 & 0 \\ \mathcal{A}_{k_{yx}} & \mathcal{A}_{b_{yx}} & -\mathcal{A}_{k_{yx}} & \mathcal{A}_{k_y} & \mathcal{A}_{b_y} & -\mathcal{A}_{k_y} \\ 0 & 0 & 0 & 0 & 0 & 0 \end{bmatrix}.$$

LQR with incomplete state observation and sensorimotor delay

To investigate possible effects of a sensorimotor delay on the simulation results, we adapted the linear optimal control model above in accordance with [2]. This was done by changing the model from one of complete state observation to one of incomplete state observation:

$$y(t) = Hx(t) + \omega(t).$$

where H is the observation matrix and $\omega(t)$ is a sensory noise term with mean 0 and covariance matrix Ω^{ω}. This formulation already implies a time delay of one time step. A sensorimotor delay of a total of 10 time steps (i.e. 100 ms, which is roughly the time to respond to a visual perturbation [3–5]) was implemented using the augmented state:

$$\tilde{x}(t) = \big[x(t), Hx(t-9), Hx(t-8), \ldots, Hx(t-1)\big].$$

An augmented observation matrix $\tilde{H}$ extracts the component $Hx(t-9)$ of $\tilde{x}(t)$

$$\tilde{H} = \big[0_{14\times14}, I_{14\times14}, 0_{14\times14}, \ldots, 0_{14\times14}\big]$$

and an augmented dynamics matrix $\tilde{A}$ removes $Hx(t-9)$, shifts the remaining sensory readings, and includes $Hx(t)$ in the next state $\tilde{x}(t+1)$

$$\tilde{A} = \begin{bmatrix} A & . & . & . & . & . & . & . & . & . \\ . & . & I_{14\times14} & . & . & . & . & . & . & . \\ . & . & . & I_{14\times14} & . & . & . & . & . & . \\ . & . & . & . & I_{14\times14} & . & . & . & . & . \\ . & . & . & . & . & I_{14\times14} & . & . & . & . \\ . & . & . & . & . & . & I_{14\times14} & . & . & . \\ . & . & . & . & . & . & . & I_{14\times14} & . & . \\ . & . & . & . & . & . & . & . & I_{14\times14} & . \\ . & . & . & . & . & . & . & . & . & I_{14\times14} \\ I_{14\times14} & . & . & . & . & . & . & . & . & . \end{bmatrix}.$$

The sensory noise terms were set to 0 except for the hand and object position and the hand and object velocity which were set to 0.01 m and to 0.1 ms^{-1} respectively. All other parameter settings were kept the same as in the model without delay. The results of the simulations are displayed in Figure S1. The model predictions for the delayed linear optimal controller are quantitatively only slightly better (i.e. explain 1%-5% more of the variance than the non-delayed version of the model) but are qualitatively very much the same. This is due to the fact that there are no unanticipated perturbations in our task and that the trials we analyse are at the end of learning when subjects already have a very good idea of the dynamics of the objects. Therefore subjects are likely to have used mostly feed-forward control rather than correcting online using the visual- and haptic feedback provided by the virtual reality setup.

Sensitivity Analysis

To analyse how sensitive both optimal control models are to the particular values of w_e and w_o chosen for the fits in the main article, we performed a sensitivity analysis on the data for condition B-low.

Dependency of R^2 on the parameters w_v, w_e and w_o (Figure S8)

We changed all three parameters from between one tenth to ten times of their initial fitted values and computed R^2 values for all the different settings. Both models (Figure S8A) are very robust to increases in w_v and w_o or decreases in w_e, which do not affect the model predictions in the range of values investigated. In contrast, increases in w_e worsen the fits slightly as the controller becomes greedier and the hand moves more directly towards the target. Similarly, decreasing w_o down to one tenth of its initial value reduces the goodness of fit only slightly as the hand path becomes straighter and the object starts missing the target. Decreases of w_v again worsen the fits slightly as the hand path becomes more curved and looses its loop mid-way (see below for more details).

Effect of w_e on model predictions (Figure S9)

To provide an intuition of how the hand path depends on w_e, the object weight w_o and the velocity weight w_v were held constant and set to the value used in the paper (linear model: $w_o = 0.05$, $w_v = 0.1$; non-linear model: $w_o = 10$, $w_v = 0.1$). w_e was varied across ten orders of magnitude (linear model: from 10^{-2} to 10^{-12} - original fit: $w_e = 10^{-8}$; non-linear model: from 2×10^{3} to 2×10^{-7} - original fit: $w_e = 2 \times 10^{-3}$).

For larger values of w_e both controllers become greedier and the hand path looses its loop and becomes straighter. Eventually the hand does not even reach the target anymore as the effort term in the cost function becomes more important than the positional and accuracy component. Reducing the effort requirement did not result in any significant changes over the range of values investigated.

Effect of w_o on model predictions (Figure S10 and S11)

The effort weight w_e and the velocity weight w_v were held constant and set to the value of the original fit (linear model: $w_e = 10^{-8}$, $w_v = 0.1$; non-linear model: $w_e = 2 \times 10^{-3}$, $w_v = 0.1$). w_o was varied across six orders of magnitude (linear model: from 5×10^{-5} to 5 - original fit: $w_o = 0.05$; non-linear model: from 0.01 to 1000 - original fit: $w_o = 10$).
Results of the simulations for the linear model are depicted in Figure S10 and for the non-linear model in Figure S11. When the object weight w_o gets smaller, the hand path becomes straighter and the object starts missing the target. Similarly, when the object weight w_o gets larger the object path becomes straighter and eventually the hand starts missing the target (non-linear model: $w_o = 10^3$).

Effect of w_v on model predictions (Figure S12 and S13)

The effort weight w_e and the object weight w_o were held constant and set to the value of the original fit (linear model: $w_e = 10^{-8}$, $w_o = 0.05$; non-linear model: $w_e = 2 \times 10^{-3}$, $w_o = 10$). w_v was varied across six orders of magnitude (linear model: from 10^{-4} to 10 - original fit: $w_v = 0.1$; non-linear model: from 10^{-4} to 10 - original fit: $w_v = 0.1$).
Results of the simulations for the linear model are depicted in Figure S12 and for the non-linear model in Figure S13. When the velocity weight w_v gets smaller, the hand and object path become more curved and the hand path looses its loop mid-way. Increasing the velocity requirement did not result in any significant changes over the range of values investigated.

LQR with model uncertainty and incomplete learning

To investigate the effects of model uncertainty and incomplete learning, we adapted the linear optimal control model above in accordance with [6]. Incomplete learning of the internal model was implemented by multiplying all entries in the A-matrix relating to the object dynamics by the scaling factor $\alpha = 0.8$ ($\alpha = 1$ would correspond to fully learned object dynamics as before):

$$A = \begin{bmatrix}
. & 1 & . & \cdots & . & . & . & \cdots \\
\alpha \times \mathcal{A}_{k_x} & \alpha \times \mathcal{A}_{b_x} & \alpha \times (-\mathcal{A}_{k_x}) & \cdots & \alpha \times \mathcal{A}_{k_{xy}} & \alpha \times \mathcal{A}_{b_{xy}} & \alpha \times (-\mathcal{A}_{k_{xy}}) & \cdots \\
. & . & . & \cdots & . & . & . & \cdots \\
. & . & . & \cdots & . & . & . & \cdots \\
. & . & . & \cdots & . & . & . & \cdots \\
. & . & . & \cdots & . & . & . & \cdots \\
. & . & . & \cdots & . & . & . & \cdots \\
. & . & . & \cdots & . & 1 & . & \cdots \\
\alpha \times \mathcal{A}_{k_{yx}} & \alpha \times \mathcal{A}_{b_{yx}} & \alpha \times (-\mathcal{A}_{k_{yx}}) & \cdots & \alpha \times \mathcal{A}_{k_y} & \alpha \times \mathcal{A}_{b_y} & \alpha \times (-\mathcal{A}_{k_y}) & \cdots \\
. & . & . & \cdots & . & . & . & \cdots \\
. & . & . & \cdots & . & . & . & \cdots \\
. & . & . & \cdots & . & . & . & \cdots \\
. & . & . & \cdots & . & . & . & \cdots \\
. & . & . & \cdots & . & . & . & \cdots
\end{bmatrix}.$$

In addition model uncertainty was introduced by adding state dependent noise to the dynamics:

$$x_{t+1} = (A + \gamma_t V)x_t + Bu_t + \sum_{i=1}^{2} \epsilon_t^i C_i u_t$$

where γ_t is a Gaussian scalar random variable with mean 0 and standard deviation 1, and V is a scaling parameter matrix which scales the variance of the model parameter uncertainty:

$$V = \begin{bmatrix} \cdot & \cdot & \cdot & \cdots & \cdot & \cdot & \cdot & \cdots \\ \sigma \times \mathcal{A}_{k_x} & \sigma \times \mathcal{A}_{b_x} & \sigma \times (-\mathcal{A}_{k_x}) & \cdots & \sigma \times \mathcal{A}_{k_{xy}} & \sigma \times \mathcal{A}_{b_{xy}} & \sigma \times (-\mathcal{A}_{k_{xy}}) & \cdots \\ \cdot & \cdot & \cdot & \cdots & \cdot & \cdot & \cdot & \cdots \\ \cdot & \cdot & \cdot & \cdots & \cdot & \cdot & \cdot & \cdots \\ \cdot & \cdot & \cdot & \cdots & \cdot & \cdot & \cdot & \cdots \\ \cdot & \cdot & \cdot & \cdots & \cdot & \cdot & \cdot & \cdots \\ \cdot & \cdot & \cdot & \cdots & \cdot & \cdot & \cdot & \cdots \\ \cdot & \cdot & \cdot & \cdots & \cdot & \cdot & \cdot & \cdots \\ \sigma \times \mathcal{A}_{k_{yx}} & \sigma \times \mathcal{A}_{b_{yx}} & \sigma \times (-\mathcal{A}_{k_{yx}}) & \cdots & \sigma \times \mathcal{A}_{k_y} & \sigma \times \mathcal{A}_{b_y} & \sigma \times (-\mathcal{A}_{k_y}) & \cdots \\ \cdot & \cdot & \cdot & \cdots & \cdot & \cdot & \cdot & \cdots \\ \cdot & \cdot & \cdot & \cdots & \cdot & \cdot & \cdot & \cdots \\ \cdot & \cdot & \cdot & \cdots & \cdot & \cdot & \cdot & \cdots \\ \cdot & \cdot & \cdot & \cdots & \cdot & \cdot & \cdot & \cdots \\ \cdot & \cdot & \cdot & \cdots & \cdot & \cdot & \cdot & \cdots \end{bmatrix}.$$

For the simulations σ was set to 0.2. The implementation is based on incomplete state observation and the sensory noise terms were set to 0 except to 0.01 m for hand and object position and to 0.1 ms^{-1} for hand and object velocity. All other parameter settings were kept the same as in the model without delay.

References

1. Todorov E, Li W (2005) A generalized iterative lqg method for locally-optimal feedback control of constrained nonlinear .... American Control Conference .

2. Todorov E, Jordan MI (2002) Optimal feedback control as a theory of motor coordination. Nat Neurosci 5: 1226–35.

3. Saunders JA, Knill DC (2005) Humans use continuous visual feedback from the hand to control both the direction and distance of pointing movements. Experimental brain research Experimentelle Hirnforschung Expérimentation cérébrale 162: 458–73.

4. Franklin DW, Wolpert DM (2008) Specificity of reflex adaptation for task-relevant variability. J Neurosci 28: 14165–14175.

5. Brenner E, Smeets JBJ (2003) Fast corrections of movements with a computer mouse. Spatial vision 16: 365–76.

6. Izawa J, Rane T, Donchin O, Shadmehr R (2008) Motor adaptation as a process of reoptimization. J Neurosci 28: 2883–91.
